# Supplementary material for: Identification of illness representational patterns and examining differences of self-care behavior in the patterns in chronic kidney disease
Source: PLoS One. 2023 Mar 31;18(3):e0283701. doi: 10.1371/journal.pone.0283701 (PMC10065431; doi:10.1371/journal.pone.0283701)
Supplement: Attachment — Submitted filename: Response_to_Reviewers.docx [file pone.0283701.s003.docx]

**Response to the Review Comments**

Please use the space provided to explain your answers to the questions above. You may also include additional comments for the author, including concerns about dual publication, research ethics, or publication ethics. (Please upload your review as an attachment if it exceeds 20,000 characters)

Overall Response: We thank the reviewers for their insightful comments. Please find a point-by-point response to the reviewers’ comments below. We believe that the revisions incorporated in the text in accordance with the reviewers’ suggestions have largely improved our manuscript and helped build a more balanced account of our research. Additional tables (A1-A4 Table) were created to respond to comments from reviewers.

The necessary information on the average eGFR values has been added to Table 1 as follows:

After revision

eGFR (mL/min/1.73 m^2^), M ± SD, N = 211ª

ª Mean values were calculated from and CKD stage in patients with available eGFR values measured at the time of the survey.

We considered and added the CKD stages and the information necessary to describe them from the reviewers' comments in Table 1.

CKD stage^b^

^b^ CKD stage was categorized by eGFR values based on the Kidney Disease Improving

Global Outcomes (KDIGO) clinical practice guidelines (45–59 [G3a], 30–44 [G3b], 15–

29 [G4], <15 [G5] ml/min/1.73 m^2^). Patients who met the criteria at recruitment but had

an eGFR ≥ 60 mL/min/1.73 m^2^　at survey were included in G3a (N=7).

*Reviewer #1: Kajiwara and Morimoto carried out a cross-sectional study examining the representational patterns present in patients with non-dialysis CKD and related self-care behavior. In a cohort of 212 patients, they identified three representational patterns with significant difference in self-care behavior related to these patterns.

This manuscript is really interesting, but I have some concerns. Briefly, my comments:
1) The Introduction and Discussion Section are not so readable because too long. I suggest to the Authors to be more concise.*

Response: Thank you for this suggestion. We have revised the Introduction to improve the conciseness of language. Several sentences that were repetitive or that did not add significant information to the research context were removed from the Introduction. From the Discussion, we have removed some statements that did not add important information. In addition, necessary information has been added in Table 1.

Thank you for your suggestion. In the following lines, we have revised the previously submitted manuscript by deleting text to make the text more concise and easier to read. The revised line numbers are as follows:

Lines 52, 54, 103, 299, 316.

For the following lines, the previously submitted text was written to be more easily interpretable:

Lines: 82, 105, 348.

*2) CKD is a disease with a broad spectrum. Is it possible to evaluate if there are any differences in representational patterns in relationship with CKD stage or CKD cause?*

Thank you for your valuable suggestion. We incorporated your comments on the cause of CKD or CKD stage, as this can make a difference in the pattern of illness representation in CKD. Initially, we had not indicated the CKD stage in the results; therefore we added information on the CKD stage in Table 1 with necessary information. Because CKD encompasses a wide range of causes, a classification of the participants is indicated as supporting information. To classify the causes of CKD, we believe that the CKD cause classification in the KDIGO practice guidelines (classification of CKD based on presence or absence of systemic disease and location within the kidney of pathologicanatomic findings in the KDIGO Clinical Practice Guidelines) is the most appropriate based on this classification (A1 Table). The causes of CKD are many and varied, and some have multiple causes. It is practically difficult to analyze and evaluate for differences in illness representational patterns by cause of CKD, and no analysis has been performed on this.

Regarding the CKD stage, as shown in A2 Table, we examined whether each cluster differed by the CKD stage. Regarding the results, G3b showed more cluster 3, G4 showed less cluster 3, and G5 showed more cluster 2 and less cluster 3. This result suggests that cluster 3 is characterized by fewer patients with severe symptoms, such as G4 and G5, and more patients with moderate symptoms, such as G3b. However, no difference has been observed in G3a, which is also moderate. We do not believe that these results are conclusive to assess that differences in the CKD stage caused differences in illness representational patterns. On the other hand, because CKD is progressive and the degree of progression varies with each patient's disease management or treatment, additional analysis was conducted to examine whether the stage of CKD makes a difference in illness representational patterns when examined in conjunction with the CKD duration (A3 Table).

In Table 1 for CKD duration, six categories were used: Less than 1 year, 1-5 years, 5-10 years, 10-15 years, 15-20 years, and more than 20 years. However, because some categories had small sample sizes, we analyzed CKD duration as four categories: Less than 1 year, 1-5 years, 5-10 years, and more than 10 years. We examined whether there were differences in the CKD stage among clusters by CKD duration, and found that in 1-5 years, G3b had fewer cluster 2 and more cluster 3, G4 had more cluster 2 and fewer cluster 3, and in more than 10 years, G3a had fewer cluster 2, G3b had more cluster 3, and G5 had more cluster 2. No differences were found in any of the CKD stages for those with less than 1 year and 5–10 years. Considering that no consistent trend was observed in any of the CKD stages, and that no differences were observed in the less than 1 year and 5–10 years of CKD duration, we do not believe that the CKD stage can be evaluated as causing differences in illness representational patterns.

*Reviewer #2: Thanks for your excellent work in which you examined what kind of representational patterns are present in patients with chronic kidney disease and the differences in self-care behavior according to them.
I wonder if there are any changes in representational patterns according to socio-demographic variables (e.g. less controllability in patients living alone vs living with family) or clinical variables (e.g. does illness representation change according to CKD duration?).
I think it would be interesting in the future to explore the relationship between illness representation and self-care behavior with other analyses, like network analyses.*

Thank you for your valuable comments. Based on your suggestion, we examined whether there are difference among clusters in CKD duration, living status, and employment status (A2, A4 Table). The results show that cluster 2 was less in 1–5 years, cluster 2 was more in 5–10 years, and cluster 3 was more in 15–20 years. However, there was no difference in the percentage of each cluster for less than 1 year, 10–15 years, and 20 years or more. There was no consistent trend by CKD duration, and it could not be determined that CKD duration made a difference in illness representational patterns. In addition, there were no differences in living status or employment status among clusters.

A1 Table. Classification of causes of CKD using KDIGO clinical guidelines (N=212)

|  | Examples of systemic diseases affecting  the kidney | | | |  |  | | |  | |  | |  | | |  | | | Examples of primary kidney diseases  (absence of systemic diseases affecting the kidney) | | | | | | | | | | | | | | | | | | |
| --- | --- | --- | --- | --- | --- | --- | --- | --- | --- | --- | --- | --- | --- | --- | --- | --- | --- | --- | --- | --- | --- | --- | --- | --- | --- | --- | --- | --- | --- | --- | --- | --- | --- | --- | --- | --- | --- |
|  |  |  |  |  |  |  | | |  | |  | |  | | |  | | |  |  |  |  |  |  |  |  |  |  |  |  |  |  |  |  |  |  |  |
|  |  | N |  | % |  |  | | |  | |  | |  | | |  | | |  | | |  | | | | N | | |  | | | % | | |  | | |
| Glomerular diseases | (1) Diabetes, systemic, autoimmune disease, | 54 | ( | 25.5 | ) |  | | |  | |  | |  | | |  | | | (5) Focal and segmental glomerulosclerosis, | | | | 14 | | | | | | ( | | | 6.6 | | | ) | | |
|  | neoplasia (including amylodosis) |  |  |  |  |  | | |  | |  | |  | | |  | | | membranous nephropathy, minimal change disease | | | | | | |  | | |  | | |  | | |  | | |
| Tubulointerstitial diseases | (2) Sarcoidosis, drugs | 4 | ( | 1.9 | ) |  | | |  | |  | |  | | |  | | |  | | | | | | |  | | |  | | |  | | |  | | |
| Vascular diseases | (3) Atherosclerosis, hypertension | 38 | ( | 17.9 | ) | |  |  | |  | |  | | |  | | | (6) ANCA-associated renal limited vasculitis | | | | | | | 2 | | | ( | | | 0.9 | | | ) | | |  |
|  | ischemia, cholesterol emboli |  |  |  |  |  | | |  | |  | |  | | |  | | |  | | |  | | | |  | | |  | | |  | | |  | | |
| Cystic and congenital diseases | (4) Polycystic kidney disease | 18 | ( | 8.5 | ) |  | | |  | |  | |  | | |  | | |  | | |  | | | |  | | |  | | |  | | |  | | |
|  |  |  |  |  |  | (7) Unkown ^a^ | | | 51 | | ( | | | 24.1 | | | ) | | |  |  | | |  | | |  | | |  | | |  | | |  |  |
|  |  |  |  |  |  | (8) Other ^b^ | | | 20 | | ( | | 9.4 | | | ) | | |  | | |  | | | |  | | |  | | |  | | |  | | |
|  |  |  |  |  |  | (9) Multiple causes ^c^ | | |  | |  | |  | | |  | | |  | | |  | | | |  | | |  | | |  | | |  | | |
|  |  |  |  |  |  | (1) and (5) | | | 1 | | ( | | 0.5 | | | ) | | |  | | |  | | | |  | | |  | | |  | | |  | | |
|  |  |  |  |  |  | (1) and (3) | | | 2 | | ( | | 0.9 | | | ) | | |  | | |  | | | |  | | |  | | |  | | |  | | |
|  |  |  |  |  |  | (1) and (8) | | | 4 | | ( | | 1.9 | | | ) | | |  | | |  | | | |  | | |  | | |  | | |  | | |
|  |  |  |  |  |  | (3) and (5) | | | 2 | | ( | | 0.9 | | | ) | | |  | | |  | | | |  | | |  | | |  | | |  | | |
|  |  |  |  |  |  | (3) and (8) | | | 2 | | ( | | 0.9 | | | ) | | |  | | |  | | | |  | | |  | | |  | | |  | | |

Note: Classification of the cause of disease was done with reference to Classification of CKD based on presence or absence of systemic disease and location within the kidney of pathologicanatomic findings in the KDIGO Clinical Practice Guidelines.

a: No diagnosis has been made and the cause is unknown.

b: Diseases not included in the Classification of CKD based on presence or absence of systemic disease and location within the kidney of pathologicanatomic findings.

(e.g. obesity-related glomerulopathy, graft-versus-host disease-related glomerulopathy)

c: Patients who have more than one of the diseases that cause CKD in the Classification of CKD based on presence or absence of systemic disease and location within

the kidney of pathologicanatomic findings or who have a concomitant disease that is included in the "Other".

CKD, Chronic Kidney Disease; KDIGO, Kidney Disease Improving Global Outcomes; ANCA, anti-neutrophil cytoplasmic antibody.

A2 Table. Differences in CKD stage and CKD duration among clusters

|  |  |  | N | cluster 1 | | | | | cluster 2 | | | | | cluster 3 | | | | |  | χ^2^ |  | p value | |
| --- | --- | --- | --- | --- | --- | --- | --- | --- | --- | --- | --- | --- | --- | --- | --- | --- | --- | --- | --- | --- | --- | --- | --- |
| CKD satge^†^, N(%) |  |  |  | N＝66 | | | | | N＝63 | | | | | N＝82 | | | | |  |  |  |  |  |
|  | G3a |  | 62 | 21 |  | ( | 33.9 | ) | 13 |  | ( | 21.0 | ) | 28 |  | ( | 45.1 | ) |  | 18.04^a^ |  |  | .006^**^ |
|  | G3b |  | 62 | 16 |  | ( | 25.8 | ) | 13 |  | ( | 21.0 | ) | 33 | ^c^ | ( | 53.2 | ) |  |  |  |  |  |
|  | G4 |  | 55 | 19 |  | ( | 34.5 | ) | 22 |  | ( | 40.0 | ) | 14 | ^d^ | ( | 25.5 | ) |  |  |  |  |  |
|  | G5 |  | 32 | 10 |  | ( | 31.3 | ) | 15 | ^c^ | ( | 46.9 | ) | 7 | ^d^ | ( | 21.8 | ) |  |  |  |  |  |
| CKD duration (years)^‡^, N (%) |  |  |  | N＝66 | | | | | N＝63 | | | | | N＝83 | | | | |  |  |  |  |  |
|  | Less than 1 year | | 23 | 10 |  | ( | 43.5 | ) | 6 |  | ( | 26.1 | ) | 7 |  | ( | 30.4 | ) |  | 20.18^b^ | |  | .  028^*^ |
|  | 1～5 |  | 77 | 30 |  | ( | 39.0 | ) | 16 | ^d^ | ( | 20.8 | ) | 31 |  | ( | 40.2 | ) |  |  |  |  |  |
|  | 5～10 |  | 43 | 10 |  | ( | 23.3 | ) | 21 | ^c^ | ( | 48.8 | ) | 12 |  | ( | 27.9 | ) |  |  |  |  |  |
|  | 10～15 | | 20 | 5 |  | ( | 25.0 | ) | 4 |  | ( | 20.0 | ) | 11 |  | ( | 55.0 | ) |  |  |  |  |  |
|  | 15～20 | | 12 | 2 |  | ( | 16.7 | ) | 2 |  | ( | 16.7 | ) | 8 | ^c^ | ( | 66.6 | ) |  |  |  |  |  |
|  | 20 or more | | 37 | 9 |  | ( | 24.3 | ) | 14 |  | ( | 37.8 | ) | 14 |  | ( | 37.9 | ) |  |  |  |  |  |

**Significant difference (p < .01), *Significant difference (p < .05).

† : CKD stage in patients with available eGFR values measured at the time of the survey (N=211) and was categorized by eGFR values based on the Kidney Disease

Improving Global Outcomes (KDIGO) clinical practice guidelines (45–59 [G3a], 30–44 [G3b], 15–29 [G4], <15 [G5] ml/min/1.73 m^2^). Patients who met the

criteria at recruitment but had an eGFR ≥ 60 mL/min/1.73 m^2^　at survey were included in G3a (N=7).

‡ : N=212.

a : Chi-square test.

b : Fisher's exact test.

c : Residue analysis showed adjusted residue values greater than 1.96.

d : Residue analysis showed that the adjusted residue value was less than 1.96.

CKD, Chronic Kidney Disease.

A3 Table. Differences in CKD stage by CKD duration among clusters (N=211)†

|  |  |  |  |  |  |  |  |  |  |  |  |  |  |  |  |  |  | χ^2, a^ |  |  |
| --- | --- | --- | --- | --- | --- | --- | --- | --- | --- | --- | --- | --- | --- | --- | --- | --- | --- | --- | --- | --- |
|  |  |  | cluster 1, N=66 | | | | | cluster 2, N=63 | | | | | cluster 3, N=82 | | | | |  | p value | |
|  |  | N | N % | | | |  | N % | | | |  | N % | | | |  |  |  |  |
| Less than 1 year^d^ | G3a | 6 | 2 |  | ( | 33.3 | ) | 1 |  | ( | 16.7 | ) | 3 |  | ( | 50.0 | ) | 3.31 |  | .769 |
|  | G3b | 9 | 5 |  | ( | 55.6 | ) | 2 |  | ( | 22.2 | ) | 2 |  | ( | 22.2 | ) |  |  |  |
|  | G4 | 5 | 2 |  | ( | 40.0 | ) | 2 |  | ( | 40.0 | ) | 1 |  | ( | 20.0 | ) |  |  |  |
|  | G5 | 2 | 1 |  | ( | 50.0 | ) | 1 |  | ( | 50.0 | ) | 0 |  | ( | 0.0 | ) |  |  |  |
| 1～5 years^e^ | G3a | 25 | 10 |  | ( | 40.0 | ) | 4 |  | ( | 16.0 | ) | 11 |  | ( | 44.0 | ) | 16.81 |  | .010^*^ |
|  | G3b | 24 | 8 |  | ( | 33.3 | ) | 1 | ^c^ | ( | 4.2 | ) | 15 | ^b^ | ( | 62.5 | ) |  |  |  |
|  | G4 | 17 | 6 |  | ( | 35.3 | ) | 8 | ^b^ | ( | 47.1 | ) | 3 | ^c^ | ( | 17.6 | ) |  |  |  |
|  | G5 | 11 | 6 |  | ( | 54.5 | ) | 3 |  | ( | 27.3 | ) | 2 |  | ( | 18.2 | ) |  |  |  |
| 5～10 years^f^ | G3a | 11 | 3 |  | ( | 27.3 | ) | 6 |  | ( | 54.5 | ) | 2 |  | ( | 18.2 | ) | 3.30 |  | .770 |
|  | G3b | 12 | 2 |  | ( | 16.7 | ) | 6 |  | ( | 50.0 | ) | 4 |  | ( | 33.3 | ) |  |  |  |
|  | G4 | 16 | 5 |  | ( | 31.3 | ) | 6 |  | ( | 37.5 | ) | 5 |  | ( | 31.3 | ) |  |  |  |
|  | G5 | 4 | 0 |  | ( | 0.0 | ) | 3 |  | ( | 75.0 | ) | 1 |  | ( | 25.0 | ) |  |  |  |
| 10 years or more^g^ | G3a | 20 | 6 |  | ( | 30.0 | ) | 2 | ^c^ | ( | 10.0 | ) | 12 |  | ( | 60.0 | ) | 14.77 |  | .022^*^ |
|  | G3b | 17 | 1 |  | ( | 5.9 | ) | 4 |  | ( | 23.5 | ) | 12 | ^b^ | ( | 70.6 | ) |  |  |  |
|  | G4 | 17 | 6 |  | ( | 35.3 | ) | 6 |  | ( | 35.3 | ) | 5 |  | ( | 29.4 | ) |  |  |  |
|  | G5 | 15 | 3 |  | ( | 20.0 | ) | 8 | ^b^ | ( | 53.3 | ) | 4 |  | ( | 26.7 | ) |  |  |  |

*Significant difference (p < .05).

† : CKD stage in patients with available eGFR values measured at the time of the survey (N=211) and was categorized by eGFR values based on the Kidney Disease

Improving Global Outcomes (KDIGO) clinical practice guidelines (45–59 [G3a], 30–44 [G3b], 15–29 [G4], <15 [G5] ml/min/1.73 m^2^). Patients who met the criteria

at recruitment but had an eGFR ≥ 60 mL/min/1.73 m^2^　at survey were included in G3a (N=7).

a : Fisher's exact test.

b : Residue analysis showed adjusted residue values greater than 1.96

c : Residue analysis showed that the adjusted residue value was less than 1.96

d : N=22

e : N=77

f : N=43

g : N=69

CKD, Chronic Kidney Disease.

A4 Table. Difference in Living status and Employment status among clusters

|  |  |  |  |  |  |  |  |  |  |  |  |  |  |  |  | χ^2, a^ |  |  |
| --- | --- | --- | --- | --- | --- | --- | --- | --- | --- | --- | --- | --- | --- | --- | --- | --- | --- | --- |
|  |  |  | N | cluster 1 | | | | cluster 2 | | | | cluster 3 | | | |  | p value | |
|  |  |  |  |  |  |  |  |  |  |  |  |  |  |  |  |  |  |  |
| Living status^b^, N (%) |  |  |  | N=66 | | | | N=62 | | | | N=82 | | | |  |  | .618 |
|  |  | Living with families | 177 | 56 | ( | 31.6 | ) | 50 | ( | 28.2 | ) | 71 | ( | 40.2 | ) | 0.96 |  |  |
|  |  | Living alone | 33 | 10 | ( | 30.3 | ) | 12 | ( | 36.4 | ) | 11 | ( | 33.3 | ) |  |  |  |
| Employment status^c^, N (%) | |  |  | N=65 | | | | N=63 | | | | N=79 | | | |  |  |  |
|  |  | Employed | 104 | 29 | ( | 27.9 | ) | 36 | ( | 34.6 | ) | 39 | ( | 37.5 | ) | 1.70 |  | .427 |
|  |  | Unemployed | 54 | 21 | ( | 38.9 | ) | 14 | ( | 25.9 | ) | 19 | ( | 35.2 | ) |  |  |  |
|  |  | Retired | 20 | 8 | ( | 40.0 | ) | 7 | ( | 35.0 | ) | 5 | ( | 25.0 | ) |  |  |  |
|  |  | Other | 29 | 7 | ( | 24.1 | ) | 6 | ( | 20.7 | ) | 16 | ( | 55.2 | ) |  |  |  |

a: Chi-square test.

b: N=210

c: N=207
